# Supplementary material for: Saccharomyces boulardii (CNCM I-745) improves intestinal damage in sepsis by remodeling intestinal flora structure
Source: Front Cell Infect Microbiol. 2025 Jul 31;15:1602792. doi: 10.3389/fcimb.2025.1602792 (PMC12350329; doi:10.3389/fcimb.2025.1602792)
Supplement: Supplementary file 1 [file DataSheet1.zip › summary/summary/8_Advanced_analyse/2_PICRUSt2_result/KEGG/Group.DW0_vs_CW0_vs_BW0_vs_AW0/PICRUSt2_KO_Group.DW0_vs_CW0_vs_BW0_vs_AW0_diff.pdf]

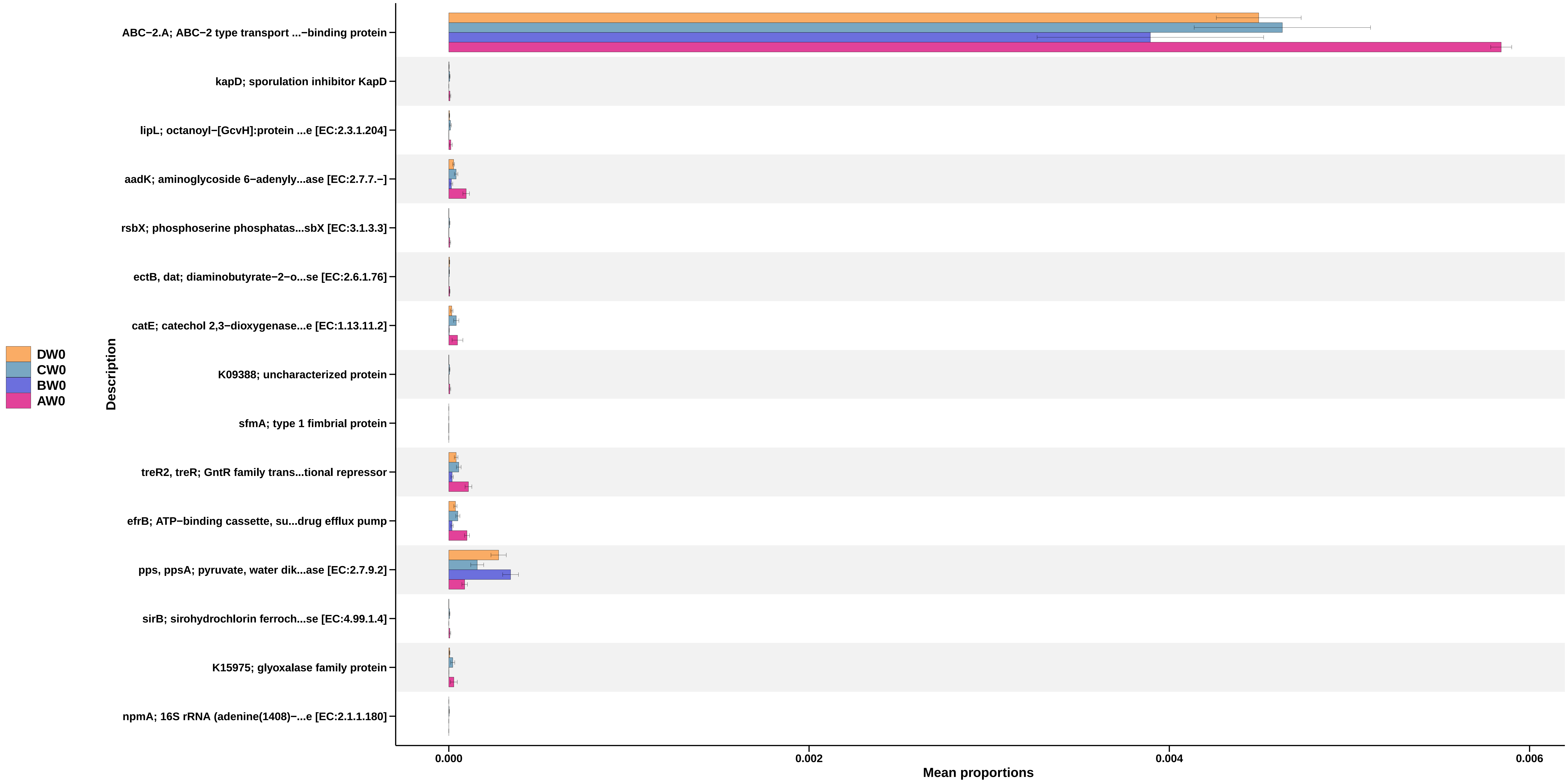
ABC−2.A; ABC−2 type transport ...−binding protein
kapD; sporulation inhibitor KapD
lipL; octanoyl−[GcvH]:protein ...e [EC:2.3.1.204]
aadK; aminoglycoside 6−adenyly...ase [EC:2.7.7.−]
rsbX; phosphoserine phosphatas...sbX [EC:3.1.3.3]
ectB, dat; diaminobutyrate−2−o...se [EC:2.6.1.76]
catE; catechol 2,3−dioxygenase...e [EC:1.13.11.2]
K09388; uncharacterized protein
sfmA; type 1 fimbrial protein
treR2, treR; GntR family trans...tional repressor
efrB; ATP−binding cassette, su...drug efflux pump
pps, ppsA; pyruvate, water dik...ase [EC:2.7.9.2]
sirB; sirohydrochlorin ferroch...se [EC:4.99.1.4]
K15975; glyoxalase family protein
npmA; 16S rRNA (adenine(1408)−...e [EC:2.1.1.180]
Description
DW0
CW0
BW0
AW0
0.000
0.002
0.004
0.006
Mean proportions
